# Application of Hybrid Extracorporeal Membrane Oxygenation for the Treatment of Subsequent Shock following Acute Respiratory Distress Syndrome Developing after Firearm Injury

**DOI:** 10.1155/2019/3120912

**Published:** 2019-12-04

**Authors:** Yahya Yildiz, Didem Melis Oztas, Mustafa Ozer Ulukan, Korhan Erkanli, Orcun Unal, Murat Ugurlucan, Halil Turkoglu

**Affiliations:** ^1^Istanbul Medipol University Medical Faculty, Department of Anesthesiology, Istanbul, Turkey; ^2^Bagcilar Training and Research Hospital, Department of Cardiovascular Surgery, Istanbul, Turkey; ^3^Istanbul Medipol University Medical Faculty, Department of Cardiovascular Surgery, Istanbul, Turkey; ^4^Yedikule Chest Diseases and Thoracic Surgery Education and Research Hospital, Cardiovascular Surgery Clinic, Istanbul, Turkey

## Abstract

The use of extracorporeal membrane oxygenation (ECMO) in acute respiratory distress syndrome (ARDS) and cardio-circulatory shock has been widely accepted. In recent years, a variety of novel and exceptional indications for ECMO have been proposed; however, experience with ECMO use in the presence of multiple penetrating injuries is limited. In this report, we present successful ECMO application in a patient with multiple firearm injuries. Veno-venous ECMO was applied for ARDS and converted to the venoarterial mode when the patient developed septic cardiomyopathy. The clinical status of the patient gradually improved, and the patient was discharged from the hospital after 24 days, successfully.

## 1. Introduction

Worldwide extracorporeal membrane oxygenation (ECMO) use has increased for the treatment of refractory patients with acute respiratory distress syndrome (ARDS). It is applied with reasonable mortality and morbidity rates to support mechanical ventilation (MV) [1]. Prolonged and high-pressure mechanical ventilation has certain risks for the pulmonary system. The veno-venous (V-V) ECMO supports patients in such critical conditions with facilitated carbon dioxide clearance and oxygenation; hence, mortality and morbidity secondary to ARDS may be attenuated [2–4].

ECMO has also use in polytrauma patients; however, experience is limited especially due to the high risk of major uncontrollable bleeding. In addition, the success of ECMO is relatively better in case of pulmonary trauma when compared with other organ systems including brain and spinal cord injuries [5].

In this report, we present our successful ECMO experience in a patient with multiple gunshot injuries.

## 2. Case Report

A 45-year-old male patient was brought to the emergency department at 01 : 00 a.m. in unconscious status with multiple gunshot injuries. He weighed 70 kg and was 168 cm tall. The patient was paraplegic. One of the bullets entered from the left arm pit and left the body from the right scapula. There was another bullet entrance from the right upper abdominal quadrant. His relatives did not present any history of previous medical issues or regular use of medications. Chest roentgenogram and computerized tomography scans indicated hemopneumothorax in the left hemithorax with destruction of the upper lobe of the left lung. In addition, the vertebral corpuses of T4 and T5 were severely destructed. There were liver and spleen injuries with intra-abdominal fluid accumulation.

**Figure 1:**
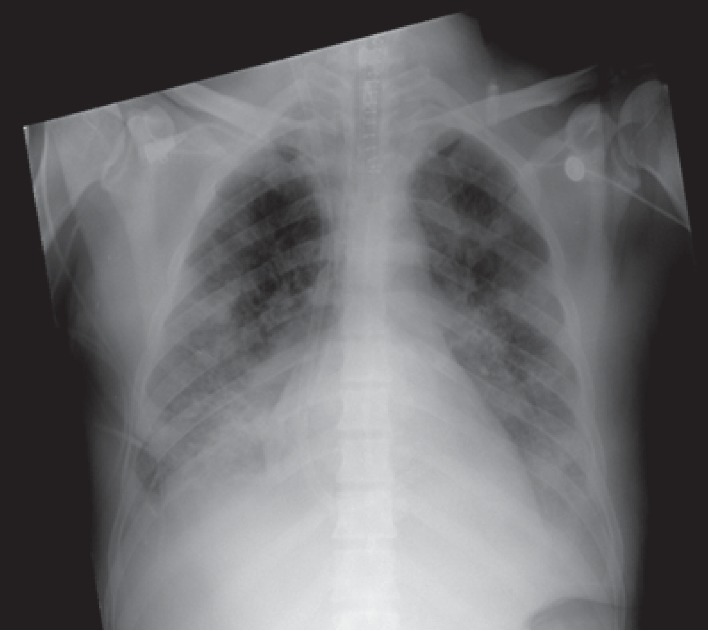
Chest roentgenogram following V-V ECMO institution with right jugular venous cannulation.

A chest tube was inserted emergently into the left hemithorax and revealed 650 ml hemorrhagic fluid drainage and resulted in expansion of the lung. He was intubated, and vertebral stabilization surgery, as well as laparotomy to treat hepatic and splenic lacerations was performed. The patient was followed at the intensive care unit, however could not be extubated. His status deteriorated on the third day (PaO_2_/FiO_2_ became 154). Endotracheal aspiration and wound cultures resulted negative. The appearance of the lungs worsened on chest roentgenograms, and we diagnosed him as ARDS according to the ARDS Berlin Criteria. Despite various mechanical ventilation and physiotherapy attempts, the status of the lungs did not improve and PaO_2_/FiO_2_ ratio became 53 and pulmonary compliance decreased to 18 ml/cmH_2_O. We decided to institute V-V ECMO with echocardiography guidance [6], despite the risk of bleeding due to multiple penetrating injuries and recent surgical treatment. ECMO flow was adjusted to 2 lt/min at a rate of 9,000 rpm, mechanic ventilator FiO_2_ of 0.6, positive end-expiratory pressure (PEEP) of 12 cmH_2_O, tidal volume of 420 ml, peak inspirium pressure of (PIP) 24 mmH_2_O, and inspiration/expiration ratio of 1/1 which were adjusted according to the blood gas analysis. However, we needed to gradually increase the flow of ECMO to 6 lt/min due to worsening of oxygenation. V-V ECMO in conjunction with mechanic ventilation provided sufficient gas exchange (Figure 1).

The patient developed septic cardiomyopathy which was confirmed with echocardiography indicating severely depressed myocardial functions (ejection fraction of 35%) on the postoperative 13th day, despite wide-spectrum antibiotherapy with vancomycin and meropenem. Although cultures were negative, white blood cell count increased to 19/uL, thrombocyte count decreased to 66/uL, CRP increased to 234 mg/L, procalcitonin increased to 8.5 ng/ml, and total bilirubin and direct bilirubin levels increased to 18 mg/dl and 16 mg/dl, respectively. His inotropic score gradually increased to 40 and we decided veno-arterio-venous (hybrid) ECMO with addition of V-A ECMO to the femoral artery and vein. The deterioration in patient's hemodynamics, echocardiography findings, and blood gas analysis are presented in Table 1.

**Figure 2:**
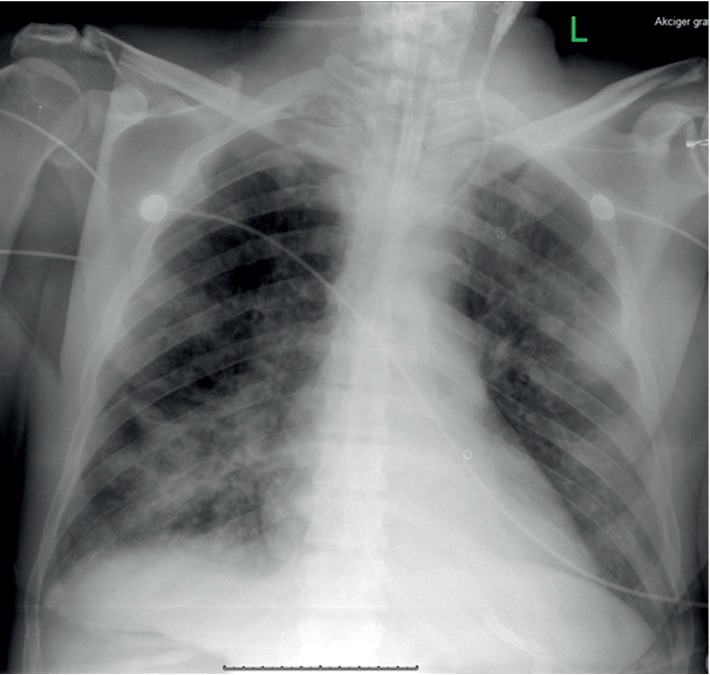
Chest roentgenogram of the patient after weaning off the ECMO.

His status gradually improved in time and he was weaned off V-A-V ECMO with rapid consecutive conversion to V-V ECMO for a few hours and then completely disconnected on the 20th day with decreased leucocyte count (9/uL), increased thrombocyte count (163/uL), decreased CRP (63), and decreased total bilirubin (3/uL) levels. His mechanical ventilation parameters were FiO_2_ 0.5, P/F 262, PEEP 8 cmH_2_O, TV 450 ml, PIP 23 cmH_2_O, I/E 1/2, and compliance 37 ml/cmH_2_O which were confirmed with chest X-ray (Figure 2). Two days after, he was in stable conditions, and percutaneous tracheostomy was performed. He was discharged from the intensive care unit and from the hospital on the 24th day and one week after, respectively.

## 3. Discussion

Acute respiratory distress syndrome is a kind of pulmonary edema which occurs due to acute inflammatory diffuse lung damage resulting in increase in pulmonary capillary permeability and fluid passage into the thoracic cavity. In this condition, the reasons that damage alveolocapillary membrane are pneumonia, sepsis in accordance with direct aspiration into the airways, and indirect heamatogenous exposure of embolism to the lungs [7].

Due to the high mortality rates of ARDS, rapid diagnosis and early mechanic ventilation are recommended in accordance with the criteria established by the American Thorax Foundation and European Intensive Care Foundation joint meeting (American-European Consensus Conference-AECC) in 1994. These criteria include an acute hypoxemia without concerning the PEEP level, the P/F ratio lesser than 200, bilateral diffuse infiltrations on chest roentgenogram, and pulmonary artery wedge pressure (PAWP) lesser than 18 mmHg. Also, the condition should not be secondary to the left heart failure. In cases of P/F ratios exceeding 200 and being lower than 300, the condition is diagnosed as acute lung injury [2].

Mechanical ventilation is a lifesaving system in patients with ARDS which enables gas exchange, whilst it may increase the pulmonary damage and increase the mortality rate. Open lung ventilation strategy with alveolar opening maneuvers and personally titrated PEEP and low tidal density support hyperventilation strategy allowing approach may attenuate mechanical ventilation-related pulmonary damage. This protocol was established in Permissive Hypercapnia, Alveolar Recruitment and Low Airway Pressure (PHARLAP) working principles, but the results remained limited [8].

According to the ARDS Berlin criteria and the clinic severity classification in mortality, the duration of ventilation and lung weight have changed drastically in such conditions. The Berlin criteria do not propose PAWP measure. Instead, it is stated that increased hydrostatic pressure is a primary source of respiratory issues, and this is outed by methods like ECMO [9]. In our case, given that the P/F value was <100 and PEEP>5, this was included into the severe class in Berlin criteria. Also, the lung compliance decreased as low as 18 ml/cmH_2_O and P/F ratio to 53. Prone position was also applied for better ventilation [10]. In order to obtain better oxygenation, we instituted V-V ECMO to the patient and adjusted mechanic ventilator parameters in order to prevent further pulmonary damage [11], despite the risk of bleeding due to multiple penetrating injuries and the surgical procedures. Severe bleeding was prevented by rigorous use of blood and blood products as well as with coagulation factors when needed. Since his condition deteriorated due to septic cardiogenic shock, we added V-A ECMO to V-V ECMO strategy in conjunction as a hybrid therapy as an established method in the literature [12]. Additional V-A ECMO was instituted through the femoral artery and vein with a 5F distal femoral line for leg perfusion [13].

Midazolam and fentanyl with appropriate sedation and adequate analgesia (VAS < 3) were provided at the intensive care unit [14]. Parenteral and low amount of enteral nutrition were started while on ECMO and continued in accordance with research that presented lower mortality with early enteral nutrition [15]. When the condition of the patient got better, we decided to wean him off hybrid ECMO. First, the femoral artery was decanulated and V-V ECMO flow was decreased to <2 lt/min, after 4 hours to <1 lt/min, and ECMO FiO2 was decreased to 25% of systematic flow. A shunt was implemented between venous and arterial lines. Four hours later, the hemodynamics and oxygenation were promising with mechanical ventilation goals, and V-V ECMO was stopped and sole mechanical ventilation was started [10]. After the cessation of ECMO, the systemic heparinization that was required during ECMO was stopped, and the patient was treated with low-molecular weight heparin for prophylaxis against pulmonary embolism or thromboembolism from the ECMO-inserted vessels [16, 17].

In conclusion, despite our single case experience, we managed our polytrauma patient with consecutive mechanical ventilation, when failed with V-V ECMO and when septic cardiomyopathy occurred in conjunction with V-A-V hybrid ECMO strategy, successfully, despite the high risk of bleeding which was prevented by rational use of blood and blood products as well as coagulation factors when needed. Our measures and successful workup prevented further damage in an already injured lung and saved the life of the patient when septic cardiomyopathy occurred without causing severe uncontrollable bleeding.

## Figures and Tables

**Table 1:** Clinical parameters of the patient.

	After the intubation	Before the V-V ECMO	Before the V-A-V ECMO	Disconnection from V-A-V ECMO
*Ventilation parameters*				
TV (ml/kg)	6–8	6	6	6–8
PEEP (cmH_2_O)	5	12–14	8–10	6
PIP (cmH_2_O)	22	38	32	26
FiO_2_	0.4–0.6	1	0.4	0.4

*Inotropes (mcg/kg/min)*				
Adrenaline	0.05	0.1	0.10	0.05
Noradrenaline	0.05	0.05	0.20	0.1
Milrinone			0.5	
Dobutamine			5	

*Blood gas analysis*				
pH	N	7.24	7.26–7.40	N
PaCO_2_ (mmHg)	N	86		N
PaO_2_ (mmHg)	86	53		
Lactate (mmol/L)	1.8–2.7	3.6–5.2	16–20	N

Urinary output (ml/kg/h)	1–3	0.3–1	0.2–1	2-3

Prone position and recruitment maneuvers	No	Yes	No	No

ECMO: extracorporeal membrane oxygenation, V-V: veno-venous, V-A-V: veno-arterio-venous, TV: tidal volume, PEEP: positive end-expiratory pressure, PIP: peak inspirium pressure, FiO_2_: fractional inspired oxygen, PaO_2_: partial oxygen pressure, PaCO_2_: partial carbon dioxide pressure, and N: normal.
